# Dr. Gilkrest's Pathological Investigation

**Published:** 1831-01-01

**Authors:** 


### XLIII.

### MILITARY HOSPITAL OF GIBRALTAR.

### DR. GILKREST ON THE GIBBALTAR FEVER.

WE have just received three numbers of the French weekly journal, *La Clinique*, for October, 1829, containing an article, by Dr. J. Gilkrest, on the late Gibraltar fever, entitled "*Exposé des Lesions Pathologiques*," *&c.* These numbers have been sent to us by a physician of our acquaintance who liberally observes, "that however warmly he may be opposed to Dr. G.'s views, as to the communicability and origin of the disease, it is quite impossible to refuse to the autbor of such an article the praise due to zealous research and practical information." We have long been in the habit of taking in La Clinique, and should perhaps have noticed the article ere this, had we not concluded that Dr. G. would probably follow up his *exposé*, by giving to the British public, in his native language, a more complete view of his large experience in this disease. In controversial medicine, which, if temperately managed, tends to the advancement of our science, we cannot too strongly deprecate bitter personalities, with which neither the public nor the profession can have any concern whatever, but which unfortunately, of late years, have but too often usurped the place of useful information, or logical deduction.

From the journal above-mentioned, we shall give a short exposition of the principal morbid appearances observed in the Gibraltar epidemic.

1. Simple redness of the gastro-intestinal mucous membrane—often perhaps the effect of congestion or stasis of the blood in the agonies of death. 2. Arborescent injection of the veins at the folds of the intestines. 3. Simple softening of the membrane above-mentioned, so as to be easily lacerable. 4. The bronchia vividly coloured in some parts of the lungs where the blood had accumulated. 5. Mollescence of the cerebral substance in persons of a certain age, with congestion of the vessels at the back part of the brain and its membranes—apparently the result of posture during and after death. 6. Vivid redness of the internal surface of the arteries from imbition. 7. Effusion of serum under the arachnoid—and the same at the basis of the brain, rogether with red points when the organ was sliced. 8. Serum in the vertebral canal, while the spinal marrow itself was sometimes very firm, sometimes in an opposite condition. 9. The internal membrane of the œsophagus altered so as to assume a rugous appearance, or even to be abraded in some instances. 10. Grey patches on the stomach—with sometimes thickening, sometimes attenuation of the mucous membrane. 11. The same in the intestinal canal. 12. The gall-bladder smaller than natural, thickened or attenuated—its lining membrane strongly inflamed—very little or no bile in this receptacle—the biliary ducts often obliterated by adhesion of their sides. 13. Generally speaking, the pericardium, heart, lining membrane of the veins, the spleen, the pancreas, the mesenteric glands, kidneys, bladder, and peritoneum were unaltered. N.B. The morbid phenomena usually found in the low fevers of Gibraltar and other countries, such as ulceration of the bowels, &c. were not observed in the late Gibraltar epidemic.

14. Yellowness (not constantly, however,) of the skin, varying from the slightest shade to the most intense orange colour—and, in cases of protracted duration, the yellow colour extended more or less to the subjacent cellular tissue, not, however, affecting the serous fluids in the pericardium or other parts. 15. Ears, fingers, and toes, more or less livid. 16. The heat of the abdominal viscera continued longer than in other fevers; but there was no peculiar fetor from the bodies of the dead, nor did they run into decomposition sooner than is usual in other fevers. 17. In cases where the disease was extremely malignant, and its course rapid, the appearances which took place immediately on the extinction of life were very remarkable. The upper half of the body quickly changed from a light yellow into a livid colour, the arms and hands often looking like those of a negro. This sudden lividity was evidently not produced by decomposition, for there was no corresponding putridity of odour. It seemed to result from transudation rather than putrefaction. In such cases the muscles appeared pale and flabby in a remarkable degree. They were extremely lacerable. The heart was in a similar condition in these malignant and rapidly fatal cases.

18. The liver, in ordinary cases, was the organ most frequently presenting well-marked morbid phenomena. Its colour differed much in the latter period of the epidemic from that which it presented in the early period;—hence the French physicians saw perhaps not more than half a dozen cases where the organ exhibited its usual characters. The usual colour of the liver was a greenish yellow or pale olive colour, approaching very nearly to that of powdered colombo-root. This colour was not confined to the surface, but extended throughout the substance of the organ. Towards the close of the epidemic it was of a brownish red colour, compared by Areguela to that of red cinchona—by M. Trousseau to that of boot tops, as formerly worn. No colouring matter was obtained from the hepatic substance by steeping, boiling, or pounding it. From livers in this state, little or no blood trickled out when they were cut into and even pressed. No traces of bile were perceptible. The organ was rarely enlarged in volume. No adhesions, abscesses, or signs of inflammation were visible. The above appearances were so constant and uniform, as to be quite characteristic of the malady in its ordinary course.

19. In cases of extraordinary malignity and rapid mortality, this change of colour in the liver was seldom well marked—but, in a considerable proportion of these cases, it was sufficiently so to shew that the organ was *passing into that state* which evinced the phenomena above-mentioned. Here the engorgement appeared to be the principal character, though it was not invariably evident. There were no traces of inflammation, adhesion, or abscess. Dr. G. and his brethren came to the conclusion that it was the *function* of the liver which was deranged in this malady rather than the structure of that organ.

20. The mucous membrane of the stomach, as was before observed, could not be considered as at all implicated in this disease, except in protracted cases, and in the same way as it is affected in almost all fevers and other diseases of some continuance. Many instances presented themselves where black vomit was unequivocal before death, and yet where no lesion of the mucous membrane of the stomach was discovered on dissection. Dr. G. comes to the conclusion now generally entertained, that the matter of black vomit is a transudation of blood, and not vitiated bile. In the small intestines, the œsophagus, and the duodenum, this fluid was sometimes found. In the large intestines a gellatinous concretion was frequently found lining the internal surface.

21. In a good many cases, there were found a number of splenitized (as the French physicians expressed it) portions of the lungs.

22. One of the most remarkable morbid appearances, but only seldom seen, was an infiltration of venous blood into the cellular tissue connecting the muscular fibres, giving to the muscles a dark or gangrenous appearance, together with considerable mollescence or lacerability.

23. A very important pathological phenomenon is noticed by Dr. G. which, he maintains, distinguishes the Gibraltar or yellow fever from the common or typhoid fevers, viz. *elliptical patches*, (commonly called the glands of Peyer) which, in the epidemic under consideration, were found on the mucous membrane of the small intestines, on the side *opposite* to that of the mesenteric line of insertion, which is just the reverse of what obtains in the common fevers, as observed by Bretonneau and various Continental pathologists.

☞ This distinction has been misunderstood and mistranslated, both by M. de Fermon, in the Bulletin des Sciences Medicales, and by the editor of the Clinique. Indeed the notice of Dr. Gilkrest's paper, in the latter journal, is inferior to that in the Bulletin; besides having omitted some notes and passages creditable to Dr. Gilkrest.

Upwards of 200 dissections were carefully made. Many dissection-wounds were received in the course of these labours, but no fatal effects resulted. No person appears to have caught the fever from the effluvia of the bodies, though many people assisted in these dissections, who never had the fever before.

---

Again we reiterate our exhortation to the parties concerned in this important investigation, to proceed in a calm and dignified manner, avoiding all acrimony of expression, and particularly personal aspersions. Wishing, with all our hearts, that we may be conducive to this amenity of manner, in the controversy, we venture, though unauthorized, to state that it was Dr. Barry himself who pointed our attention to Dr. Gilkrest's able paper, bearing most liberal testimony to its merits and fidelity. May this prove an omen of returning good feeling, and the dismission of personal irritation in the pursuit of science and truth.—*Ed.*

